# Density‐dependent space use affects interpretation of camera trap detection rates

**DOI:** 10.1002/ece3.5840

**Published:** 2019-11-22

**Authors:** Kate Broadley, A. Cole Burton, Tal Avgar, Stan Boutin

**Affiliations:** ^1^ Department of Biological Sciences University of Alberta Edmonton AB Canada; ^2^ Department of Forest Resources Management and Biodiversity Research Centre University of British Columbia Vancouver BC Canada; ^3^ Department of Wildland Resources Utah State University Logan UT USA

**Keywords:** animal density, animal movement, camera trap, home range, imperfect detection, population ecology, spatial ecology, wildlife monitoring

## Abstract

Camera traps (CTs) are an increasingly popular tool for wildlife survey and monitoring. Estimating relative abundance in unmarked species is often done using detection rate as an index of relative abundance, which assumes that detection rate has a positive linear relationship with true abundance. This assumption may be violated if movement behavior varies with density, but the degree to which movement behavior is density‐dependent across taxa is unclear. The potential confounding of population‐level relative abundance indices by movement would depend on how regularly, and by what magnitude, movement rate and home‐range size vary with density. We conducted a systematic review and meta‐analysis to quantify relationships between movement rate, home‐range size, and density, across terrestrial mammalian taxa. We then simulated animal movements and CT sampling to test the effect of contrasting movement scenarios on CT detection rate indices. Overall, movement rate and home‐range size were negatively correlated with density and positively correlated with one another. The strength of the relationships varied significantly between taxa and populations. In simulations, detection rates were related to true abundance but underestimated change, particularly for slower moving species with small home ranges. In situations where animal space use changes markedly with density, we estimate that up to thirty percent of a true change in relative abundance may be missed due to the confounding effect of movement, making trend estimation more difficult. The common assumption that movement remains constant across densities is therefore violated across a wide range of mammal species. When studying unmarked species using CT detection rates, researchers and managers should explicitly consider that such indices of relative abundance reflect both density and movement. Practitioners interpreting changes in camera detection rates should be aware that observed differences may be biased low relative to true changes in abundance. Further information on animal movement, or methods that do not depend on assumptions of density‐independent movement, may be required to make robust inferences on population trends.

## Graphical abstract

Animal movement rate and home range size are density‐dependent across a wide range of taxa. This potentially confounds interpretation of detection indices from camera trap surveys. When studying unmarked species using detection rates, researchers and managers should explicitly consider that such indices of relative abundance reflect both density and movement.

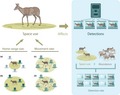

## 1 INTRODUCTION

Accurate abundance estimation is at the core of wildlife management, and camera traps (CTs) are an increasingly popular monitoring tool (Burton et al., 2015; O'Connell, Nichols, & Karanth, 2011). Camera traps are triggered by a temperature differential and movement across their detection zone, thus capturing images of warm‐blooded animals that pass by. Compared to traditional live trapping, camera trapping is less expensive and less invasive (Kucera & Barrett, 2011), and it can be used for the simultaneous collection of data on multiple species (Burgar, Burton, & Fisher, 2019; Tobler, Carrillo‐Percastegui, Leite Pitman, Mares, & Powell, 2008). This makes CTs very attractive to those wishing to conduct large‐scale, multispecies monitoring programs. The camera trapping technique has been particularly successful in the study of large species whose coat patterns allow individuals to be identified. Identification of such “marked” species allows for standard capture–recapture methods to be applied (Karanth & Nichols, 1998), and accurate density estimation of such populations has been further improved with the development of spatially explicit capture–recapture methods (Efford, 2004; Royle, Chandler, Sollmann, & Gardner, 2014).

However, most species cannot be reliably identified individually by unique markings. This presents a challenge to those wishing to monitor these populations, and so scientists and managers must rely on alternatives to standard capture–recapture methods for estimation of density or abundance. Indices that document relative changes are one such alternative to more formal methods of density estimation (Johnson, 2008; Williams, Nichols, Conroy, & Michael, 2002). In fact, indirect measures of population trends, like relative abundance and presence/absence, represent the most common ways CT data are analyzed, with relative abundance being reported in over 40% of studies (Burton et al., 2015). Relative abundance indices typically consist of detections standardized by effort (sampling duration): If camera and site conditions are appropriately controlled for, variation in detection rate will be driven solely by animal‐specific factors, and if space use remains consistent, changes in population density should be the primary factor responsible for changes in detection rate. That is, if individual encounter rates (i.e., the probability that a given individual will encounter a camera per unit time) remain constant across space and time, the overall detection rate serves as a valid index of abundance, and the change in detection rate over space or time is linearly related to the change in population density (O'Brien, 2011). Detection‐based relative abundance indices have been employed in many published studies (e.g., Bengsen, Leung, Lapidge, & Gordon, 2011; Carbone et al., 2001; O'Brien, 2011; O'Brien, Kinnaird, & Wibisono, 2003), while others have noted their frequent use within the gray literature of wildlife management reports and conservation strategies (Sollmann, Mohamed, Samejima, & Wilting, 2013).

**Figure 1:**
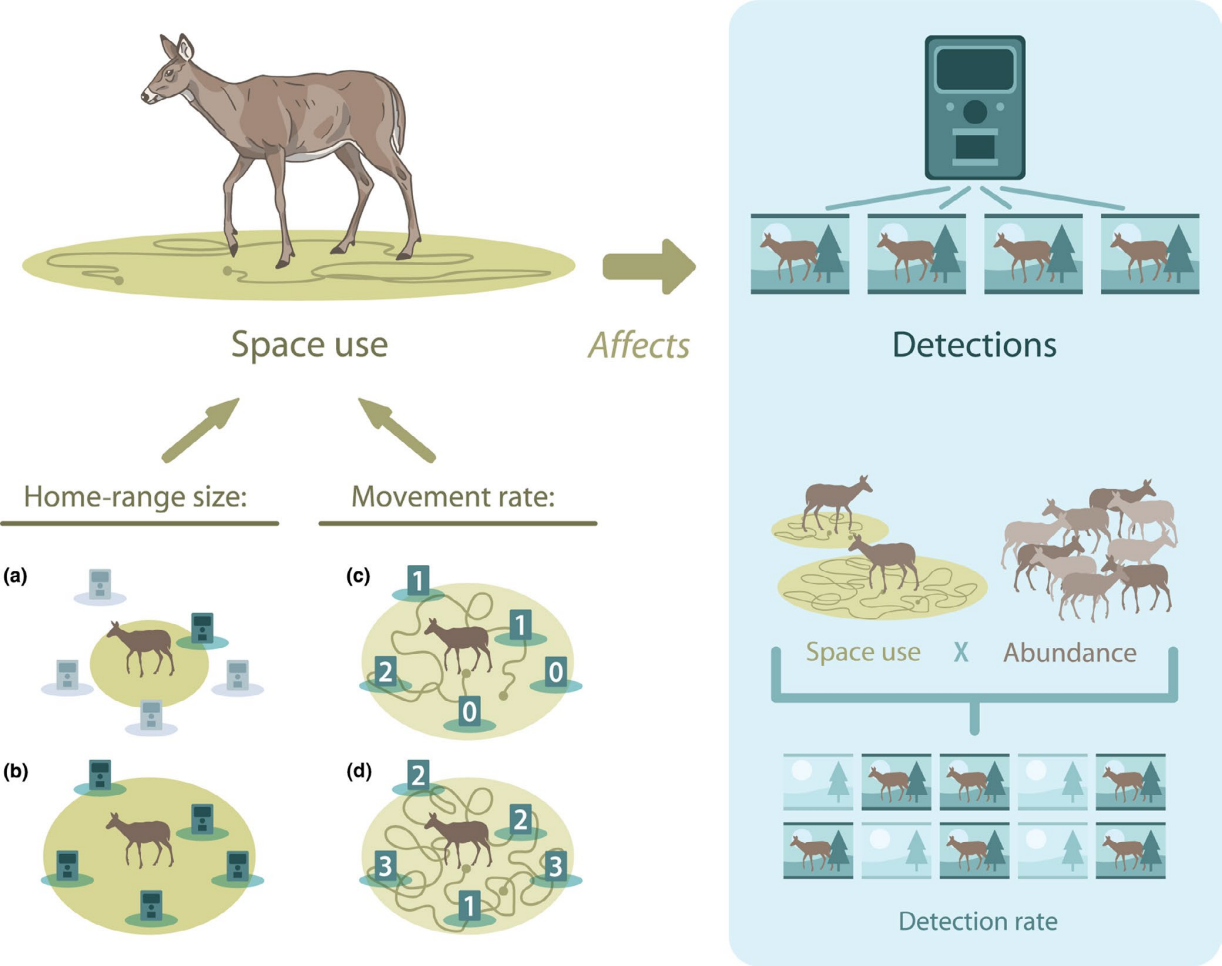
Space use is one factor affecting detections on CTs. Here, two aspects of space use are considered: home‐range size and movement rate. (a) Individuals with smaller home ranges overlap fewer cameras on average (and those with very small ranges may not overlap any cameras). (b) Individuals with larger home ranges overlap and can be detected at more cameras. (c) Individuals that move through their home range more slowly will take longer to encounter cameras. (d) Individuals that move more quickly encounter cameras more frequently within the same span of time and produce more detections. Thus, detection rate is a function of both the relative abundance of the population, as well as the space use of the animals that make up that population

Abundance, however, is not the only factor that influences detection rates. Along with factors like the camera's settings, the size of its detection zone, vegetation cover, etc. (Burton et al., 2015), animal space use will also influence detectability (Neilson, Avgar, Burton, Broadley, & Boutin, 2018; Stewart, Fisher, Burton, & Volpe, 2018; Figure 1). The core assumption of constant encounter probability is thus likely untrue (e.g. Harmsen, Foster, Silver, Ostro, & Doncaster, 2010; Sollmann, Mohamed, et al., 2013). Violation of this assumption poses a problem for the use of detection rates as an index of relative abundance when encounter probability covaries with density (Harmsen et al., 2010; Jennelle, Runge, & MacKenzie, 2002). Wide‐ranging individuals are likely to encounter more cameras, but rarely encounter the same camera multiple times, compared to individuals with more spatially concentrated space use. When passive traps like cameras are distributed randomly over large spatial scales, both the mean encounter rate and time to first encounter are strongly affected by animal movement speed, particularly given the small detection zone typical of camera traps (Gurarie & Ovaskainen, 2013). If home‐range size and movement rate are density‐dependent, this could obscure true change in abundance inferred from a relative abundance index. This is particularly problematic if home range and movement rate are negatively correlated with density. At best, a relative abundance index will underestimate the true change in abundance. At worst, increased movement rates and home‐range sizes at low densities could result in a few individuals producing more total detections than would a higher density population.

Habitat availability and quality is an obvious common driver of negative density‐dependent movement and home‐range size. Conceptually, home‐range size is an emergent property of individual movements, where the duration spent in a location and/or the frequency at which a location is revisited affects the overall space‐use pattern of an animal (Van Moorter, Rolandsen, Basille, & Gaillard, 2016). Higher‐quality habitats support higher population densities, with each individual in those populations occupying a smaller home range and moving less (due to higher resource density and/or territoriality; Avgar, Mosser, Brown, & Fryxell, 2013; Fryxell et al., 2008; Harestad & Bunnell, 1979; Owen‐Smith, Fryxell, & Merrill, 2010). Conversely, individual space use may be driven by the intensity of intraspecific competition, with movement speed and range positively related to population density due to conspecific repulsion (e.g., Kuefler, Avgar, & Fryxell, 2012). Whichever is the case, covariation between movement, home‐range size, and density is expected to be the norm across most animal populations, and one would expect the assumption of density‐independent movement to be frequently violated.

This paper aims to quantify how home‐range size, movement rate, and density covary across mammalian taxa, and to explore the effect, this covariation might have on CT‐based relative abundance estimates. In order to quantify the relationships between movement rate, home‐range size, and density, we conducted a systematic meta‐analysis of published studies that have reported any two of these parameters in any terrestrial mammalian taxa. We then used simulations to quantify the magnitude of the expected effect of density‐dependent movement on interpretation of CT detection rates. We chose space‐use scenarios that span the range of variation found in the meta‐analysis, so as to reveal the full extent by which movement could confound relative abundance estimates from detection rates.

## 2 METHODS

### 2.1 Literature search

We searched for relevant articles in the Web of Science database using the following search terms: (“movement” OR “distance travelled” OR “distance moved” OR “speed” OR “activity”) AND (“population density” OR “abundance” OR “home range size” OR “territory size”) AND (“mammal” OR “mammalian”). The search was conducted in 2015 and restricted to English language papers published from 2005 to 2015. Studies on any terrestrial mammalian taxa were considered. We retained those studies that reported at least two of either movement rate, (relative) density/abundance, or home‐range size (Table S1). The list was further restricted to only papers that reported these parameters for at least two different “populations” (defined spatially or temporally, i.e., the same population in different years); this allowed us to control for variation in methodology (Table S1) by using only within‐study comparisons made with consistent methods. Excluded were any studies that used a telemetry location interval greater than 24 hr to calculate movement rates, or for which one of the parameters was confounded by the other parameter (e.g., a population density calculated as the number of home ranges that could fit in the study area would not be considered valid). Also omitted were studies that only compared single individuals as well as studies that compared islands to mainland areas so as to exclude situations of unusually constrained spatial geography.

For the resulting set of papers, the species, taxonomic order, parameters measured, and methods used to measure the relevant parameters were recorded. We defined movement rate as the average displacement between consecutively observed positions divided by the time interval between these positions. Movement rate is thus a relative measure of speed as fix rate varies between studies, and the lower the fix rate the more underestimated the movement rate compared to the true speed of the animal (Street, Avgar, & Börger, 2018). Terminology for this parameter varies widely across movement studies, so the methods of each individual study were examined carefully. Only the parameter whose description matched the definition for movement rate given above was selected for analysis, regardless of the terminology originally applied in the source.

### 2.2 Magnitude of variation

We considered three relationships: movement rate versus density, home‐range size versus density, and home‐range size versus movement rate. To compare the relationships between studies and systems, we calculated the ratio of the higher value to the lower value for the populations in the study. The population with the lowest value for the predictor variable (either population density or home‐range size) was considered the “reference” population, and we then calculated the increase in that parameter from the “reference” to the other population. For studies with more than two populations, we used the populations that offered the greatest difference across the predictor variable range. We then calculated the value for the response variable (either home‐range size or movement rate) as the percentage of its value in the “reference” population (such that 100% represents no change in value). This was done to quantify and visualize the actual magnitudes of changes in these variables, rather than simply a positive or negative correlation.

### 2.3 Meta‐analysis

A meta‐analysis calculates an overall effect size from a set of standardized effect sizes, thereby determining the magnitude of the effect of one variable on another (Rosenthal & DiMatteo, 2001). Given that all of the parameters analyzed (density, movement rate, and home‐range size) are continuous, the correlation coefficient (*r*) was chosen as the most appropriate measure of effect size. In this context, the overall correlation coefficient represents the strength and direction of the association between a given pair of parameters. Correlation coefficients of individual studies were obtained either from direct reporting, or from converting other test statistics given. When a test statistic other than r was reported (e.g., *t*, *χ* ^2^ *, F*, and *U*), it was changed using standard conversion formulae into the Fisher's Z transformation of *r* (Rosenthal & DiMatteo, 2001). For example, a *t* statistic from a study comparing two mean movement rates can be converted into a correlation coefficient using the formula $r=\sqrt{\frac{t^{2}}{t^{2}+df}}$. Once standardized, these effect sizes (weighted by the inverse of their variances) were analyzed together using a random effects model to determine the overall strength and direction of the effect. Unlike a fixed effects model which assumes there is only one true effect size (and that all observed differences in effects are due to sampling error), a random effects model allows for the event that the true effect size varies from study to study. A random effects model is preferable for ecological phenomena because it accounts for variance in true effect size across systems and allows generalization to studies outside of the meta‐analysis (Cooper et al., 2009; Rosenthal & DiMatteo, 2001). Cochran's *Q* test for heterogeneity was used to assess the significance of variation in effect sizes across studies in the meta‐analysis. Significant heterogeneity indicates that sampling error alone cannot explain variance in effect sizes.

### 2.4 Animal simulations

The effect of density‐dependent movement on camera detection rates was examined using a set of computer simulations. To make our results as general as possible, we varied only two attributes of animal space‐use dynamics; the degree of attraction to a focal point (giving rise to stable home ranges) and the rate of positional shifts (speed). Animal movement was simulated using a “stepping‐stone” approach, in which individuals move independently across a regular grid of 10^7^ hexagonal cells (Avgar, Potts, Lewis, & Boyce, 2016; see also Neilson et al., 2018). Assuming that cell area is 100 m^2^, our domain represents a 1,000 km^2^ study area. The domain was wrapped around a torus, thus eliminating any edge effects. Individuals were given random home‐range centers within this domain. The movement model comprises a discrete biased random walk, where during each time step, *t*, an individual could move to an adjacent hexagonal cell, or remain in place. The position of the animal at the next time step is described by a truncated redistribution kernel of the form:

(1) $$p\left(x_{t+\tau }=x\right)=\frac{I\left(\|x-x_{t}\|\leq 1\right)\exp\left(-\alpha \|x-x_{t}\|-\beta \|x-x^{\prime }{\|}^{2}\right)}{\sum I\left(\|x-x_{t}\|\leq 1\right)\exp\left(-\alpha \|x-x_{t}\|-\beta \|x-x^{\prime }{\|}^{2}\right)}.$$

in which *x_t_*_+_ *_τ_* represents the cell to be occupied by the animal at the next time step, *τ* (=10 s) is the time step's duration, *x*′ is the individual's home‐range center, *I* is an indicator function valued at 1 or 0 based on the immediately following expression, *α* is the movement cost, and *β* is a parameter determining attraction to the home‐range center. This model of animal movement allows for a realistic simulation of animal paths, as observed patterns in speed and home‐range size are an emergent property of the movement process, rather than the result of imposed boundaries. By altering the movement cost (*α*) and the home‐range attraction (*β*) parameters, different space‐use patterns can be simulated. The movement cost parameter (*α*) is easily converted into a more biologically relevant parameter: the probability that an animal will move during a time step, $\mu ={\left[1+\left(e^{\alpha }/6\right)\right]}^{-1}$. Modifying this probability in turn modifies the movement rate. The steady‐state home range (i.e., the utilization distribution the animal will eventually produce if given enough time) takes the form of a bivariate isotropic normal distribution with variance given by ${\left(4\cdot \beta \right)}^{-1}$. The steady‐state home‐range size is thus approximately 723.4∙*β* ^−1^ m^2^, if 99% of the utilization distribution is to be included (see Signer, Fieberg, & Avgar, 2017). In summary, our model simulates space‐use patterns by a population of solitary nonterritorial animals with independently and randomly distributed home‐range centers. We note that this model is a first step in modeling realistic animal movements, with many potential complexities remaining to be explored in future research (e.g., territoriality, herding, and habitat selection).

Two contrasting movement scenarios were chosen to evaluate the relationship between animal density, speed, home‐range size, and detection rate: one with fast movement rates and large home ranges, and one with slow movement and small home ranges. These scenarios were chosen to capture the relationship found in the meta‐analysis, where movement rate and home‐range size are both negatively correlated with density and positively correlated with one another. The fast, large home‐range population had a steady‐state home‐range size of 100 km^2^ and μ=1 (mean realized speed of approx. 180 m/hr). The slow, small home‐range population had a steady‐state home‐range size of 1 km^2^ and μ=0.1 (mean realized speed of approx. 57 m/hr). These scenarios were chosen to reflect a range in movement that would encompass the possible variation found in real populations. For instance, movement rate can more than triple over a 25‐fold change in home‐range size (chacma baboon, Hoffman & O'Riain, 2012). By covering the possible range in variation found in nature, the simulation can represent a “worst‐case scenario” and is able to reveal the full extent to which space use can confound population estimates.

Individuals were allowed to move for 3,153,600 time steps (1 year in total). To remove effects of the initial conditions, only the last 1,576,800 steps (6 months) were used for analysis. One thousand random cells in the domain were designated as CT sites, such that the presence of an individual within that cell during a time step was counted as a detection on that camera. This represents an ideal scenario for camera trapping: an intense, random sampling regime with perfect detections (given that an animal is preset with the cell during the study). One hundred individuals were simulated for each movement scenario. The final output was a record of how many times a given animal was detected at each camera location over the duration of the simulation. To get the detection rate for a given camera, the detections from all One hundred individuals at that camera were summed and then divided by the study duration. In order to simulate variation in density, 25, 50, and 75 individuals were subsampled from the full population of 100, and their detections at a given camera location summed. The mean detection rate in all cases was then obtained by averaging the detection rates of all 1,000 cameras. A Tukey HSD test was used to test for significant differences between mean detection rates.

## 3 RESULTS

### 3.1 Meta‐analysis

The literature review resulted in 42 studies covering 43 species from six orders, with carnivores (13), primates (13), rodents (10), and ungulates (7) (see Appendix S1 for detailed study information).

**Figure 2:**
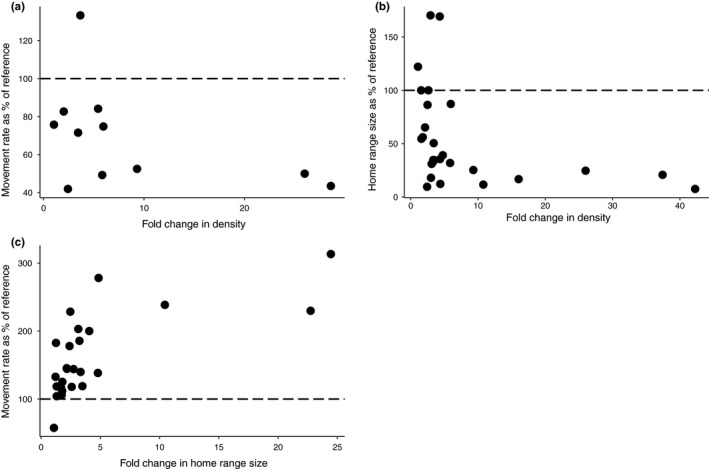
(a) Movement rate in a higher density population expressed as a percentage of the lower density population's movement rate (i.e., reference value). 100% represents no change in movement rate. Points that fall below the 100% line indicate a drop in movement rate over a given change in density; points that fall above the 100% line indicate an increase in movement rate over a given change in density. *N* = 11 studies. (b) Home‐range size in a higher density population expressed as a percentage of the lower density population's home range (i.e., reference value). *N* = 26 studies. (c) Movement rate in a population with larger home ranges expressed as a percentage of the smaller home‐range population's movement rate (i.e., reference value). *N* = 25 studies

Movement rates were included in only 11 studies that reported density (Figure 2a), and in 10 of these, the higher density population exhibited a lower movement rate. There appeared to be a nonlinear relationship between the magnitude of the decrease in movement rate and the magnitude of the density change. Movement rates decreased by a maximum of just over 50% despite changes in density of up to 26‐fold. Most density changes were on the order of <10‐fold.

Increases in density were associated with decreases in home‐range size in 22 of 27 cases (Figure 2b). While the most pronounced decrease was an approximate 90% reduction in home‐range size over a 2.4‐fold increase in density (coyotes; Wilson & Shivik, 2011), there was a nonlinear relationship between the magnitude of the decrease in home range and the magnitude of the density change (up to 42.2‐fold). Three species, (white‐tailed deer; Webb, Hewitt, & Hellickson, 2007; chamois; Brambilla, Bocci, Ferrari, & Lovari, 2006; and degu; Quirici et al., 2010) exhibited an increase in home‐range size with density.

An increase in home‐range size was usually associated with an increase in movement rate (Figure 2c). Movement rates increased to a maximum of just over 300% of the reference value while home‐range sizes differed by up to 25‐fold. Most home‐range size changes, however, were on the order of <5‐fold.

**Figure 3:**
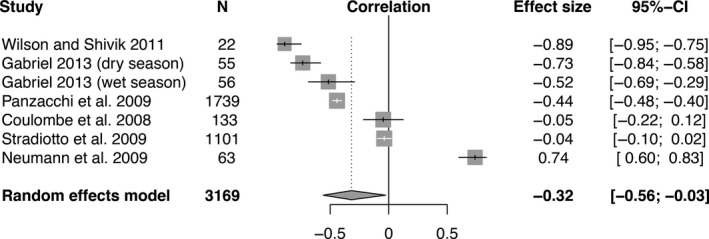
Forest plot indicating the mean effect size (correlation coefficient) of studies that reported movement rates across different densities. The effect size is significant if its 95% confidence interval (the black bar) does not overlap zero. Negative correlation coefficients indicate that movement rates are slower at higher densities. The diamond indicates the overall effect size as determined by a random effects model

**Figure 4:**
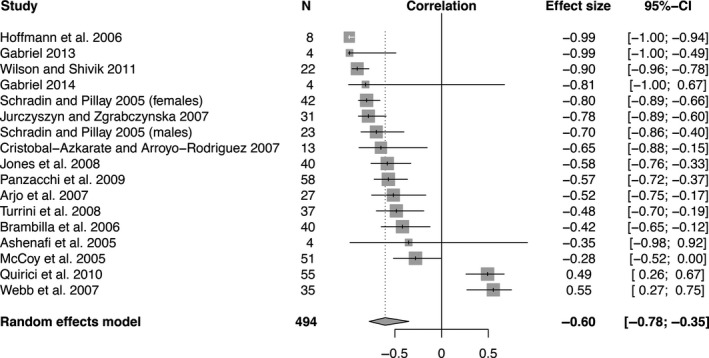
Forest plot indicating the mean effect size (correlation coefficient) of studies that reported home‐range sizes across different densities. The effect size is significant if its 95% confidence interval (the black bar) does not overlap zero. Negative correlation coefficients indicate that home ranges are smaller at higher densities. The diamond indicates the overall effect size as determined by a random effects model

**Figure 5:**
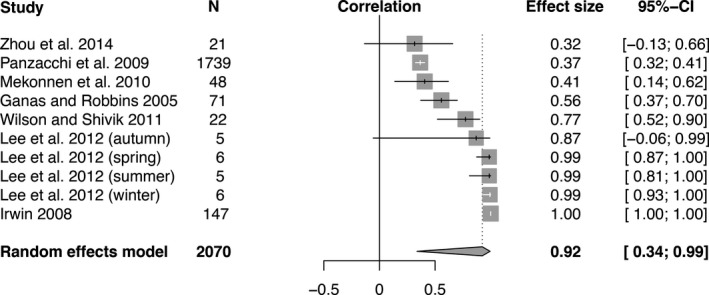
Forest plot indicating the mean effect size (correlation coefficient) of studies that reported movement rates across populations with different home‐range sizes. The effect size is significant if its 95% confidence interval (the black bar) does not overlap zero. Positive correlation coefficients indicate that movement rates are slower at when home ranges are smaller. The diamond indicates the overall effect size as determined by a random effects model

Meta‐analysis of effect sizes using a random effects model revealed a significant negative relationship between movement rate and density (Figure 3, *r* = −.32, *p* = .03). Density and home‐range size were also significantly negatively correlated (Figure 4, *r* = −.60, *p* < .0001). Furthermore, there was a significant positive relationship between movement rate and home‐range size (Figure 5, *r* = .92, *p* = .012). Effect sizes were significantly heterogeneous across studies for all three comparisons: movement rate versus density (Figure 3, *Q*_6_ = 277.99, *p* < .0001), home‐range size versus density (Figure 4, *Q*_16_ = 172.82, *p* < .0001), and movement rate versus home‐range size (Figure 5, *Q*_9_ = 2185.67,* p* < .0001).

### 3.2 Animal simulations

**Figure 6:**
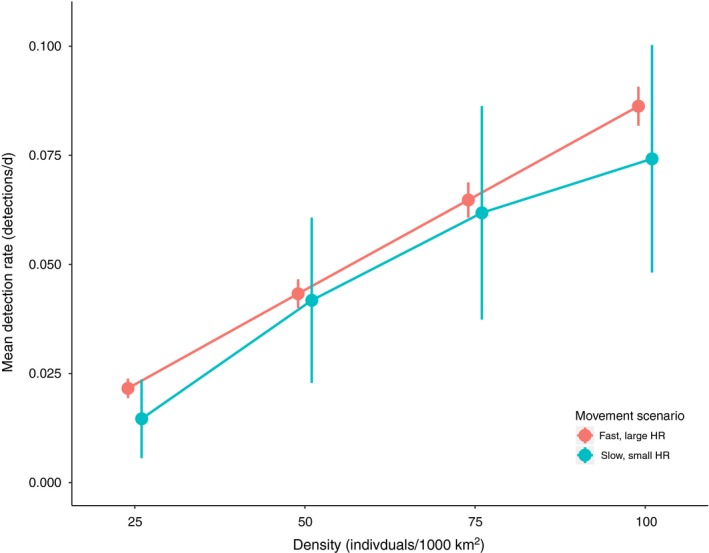
Mean detection rate under different densities and movement scenarios. Movement scenarios represent home‐range sizes and movement rates that covary and encompass the possible range in behavior as seen in the meta‐analysis. Populations of 25, 50, 75, and One hundred individuals were simulated in a 1,000 km^2^ domain. The fast movement, large home‐range scenario is indicated in red (realized speed 180 m/hr, steady‐state home range 100 km^2^) while the slow movement, small home‐range scenario is indicated in blue (realized speed 57 m/hr, steady‐state home range 1 km^2^). Detection rates represent the mean detection rate across 1,000 randomly placed cameras over a time span of 6 months. Error bars represent 95% confidence intervals

Under the fast, large home‐range scenario (realized speed 180 m/hr, steady‐state home range 100 km^2^), the mean detection rate changed linearly, such that a change in density was associated with a proportional change in detection rate. For instance, the mean detection rate produced by One hundred individuals/1,000 km^2^ (Figure 6, mean = 0.086 detections/day, *SD* = 0.072) was approximately double the mean detection rate produced by 50 individuals/1,000 km^2^ (Figure 6, mean = 0.043 detections/day, *SD* = 0.053).

In contrast, the slow, small home‐range scenario (realized speed 57 m/hr, steady‐state home range 1 km^2^), produced a nonlinear trend in detection rate, where detections increased at a diminishing rate as density increased (Figure 6). Differences in detection rates between populations of different movement scenarios were only evident across relatively large density changes. For instance, the detection rate of the fast, large home‐range population with 25 individuals/1,000 km^2^ was not significantly different from the slow, small home‐range population unless it had a density of 75 individuals/1,000 km^2^ or greater (Table 1).

**Table 1:** Pairwise tests (Tukey HSD) of differences (and 95% confidence limits) in detection rates between simulation scenarios that compared fast versus slow movement rates and small versus large home‐range sizes across four population densities (25, 50, 75, and One hundred individuals per 1,000 km^2^)

Treatment 1	Treatment 2	Difference	Lower limit	Upper limit	*p* value
fast_large:25	fast_large:50	0.0217	−0.0109	0.0542	.4681
fast_large:25	fast_large:75	0.0431	0.0106	0.0757	.0015*
fast_large:25	fast_large:100	0.0646	0.0321	0.0972	<.0001*
fast_large:25	slow_small:25	−0.0070	−0.0395	0.0256	.9981
fast_large:25	slow_small:50	0.0202	−0.0124	0.0527	.5660
fast_large:25	slow_small:75	0.0402	0.0077	0.0728	.0045*
fast_large:25	slow_small:100	0.0526	0.0200	0.0851	<.0001*
fast_large:50	fast_large:75	0.0215	−0.0111	0.0540	.4824
fast_large:50	fast_large:100	0.0430	0.0104	0.0755	.0016*
fast_large:50	slow_small:50	−0.0015	−0.0341	0.0310	1.0000
fast_large:50	slow_small:75	0.0185	−0.0140	0.0511	.6698
fast_large:50	slow_small:100	0.0309	−0.0016	0.0634	.0769
fast_large:75	fast_large:100	0.0215	−0.0110	0.0540	.4799
fast_large:75	slow_small:75	−0.0029	−0.0355	0.0296	1.0000
fast_large:75	slow_small:100	0.0094	−0.0231	0.0420	.9878
fast_large:100	slow_small:100	−0.0120	−0.0446	0.0205	.9521
slow_small:25	fast_large:50	0.0287	−0.0039	0.0612	.1316
slow_small:25	fast_large:75	0.0501	0.0176	0.0827	.0001*
slow_small:25	fast_large:100	0.0716	0.0391	0.1042	<.0001*
slow_small:25	slow_small:50	0.0271	−0.0054	0.0597	.1832
slow_small:25	slow_small:75	0.0472	0.0147	0.0797	.0003*
slow_small:25	slow_small:100	0.0596	0.0270	0.0921	<.0001*
slow_small:50	fast_large:75	0.0230	−0.0096	0.0555	.3883
slow_small:50	fast_large:100	0.0445	0.0119	0.0770	.0009*
slow_small:50	slow_small:100	0.0324	−0.0001	0.0650	.0515
slow_small:50	slow_small:75	0.0201	−0.0125	0.0526	.5728
slow_small:75	fast_large:100	0.0244	−0.0081	0.0570	.3073
slow_small:75	slow_small:100	0.0124	−0.0202	0.0449	.9449

* signifies statistical significance at *p* < 0.01.

Comparisons between movement scenarios also inaccurately represented the true change in density. For example, the slower, smaller home‐range scenario at One hundred individuals/1,000 km^2^ (Figure 6, mean = 0.074 detections/day, *SD* = 0.42) had a detection rate approximately 1.7 times greater than the faster, larger home‐range scenario with 50 individuals/1,000 km^2^ (Figure 6, mean = 0.043 detections/day, *SD* = 0.053).

## 4 DISCUSSION

As predicted, our meta‐analysis indicated that higher population densities were associated with significantly slower movement rates and smaller home ranges across multiple species. This relationship violates the common assumption that changes in detection rates reflect only changes in relative abundance and not changes in movement, and has important implications for wildlife monitoring programs that rely on unvalidated detection rate indices from CT sampling. Because a population in decline would produce more detections per individual through increased movement, one may falsely conclude that the population is stable (or at best declining less rapidly).

Despite overall significant negative relationships between density and both movement rate and home‐range size, there were some exceptions. These cases exhibited a positive correlation between density and either movement rate or home‐range size. Such a relationship is also problematic for detection rate indices, as it would lead to overestimates of changes in relative abundance. At least one of these cases was explained by greater landscape patchiness in resource‐rich areas (e.g., Brambilla et al., 2006). Delayed density‐dependence may also be the cause, as when a decrease in resources has a delayed effect on population density (e.g., Quirici et al., 2010). Dispersal dynamics are another potential cause of positive density‐dependent movement. High densities can drive some individuals to disperse as a way to avoid inbreeding, predators, parasites, exploitative competition, or interference competition (Bowler & Benton, 2005), although positive density‐dependent dispersal is more commonly observed in experimentally manipulated populations rather than site‐to‐site or year‐to‐year comparisons (Matthysen, 2005).

While the predicted trends of density‐dependent space use were common, the degree to which the relationship between density and movement affects interpretation of camera data depends on the magnitude. Significant heterogeneity between effect sizes observed in the meta‐analysis indicates that no singular magnitude of effect can be expected across different taxa or systems. Movement rate and home‐range size may change dramatically with density, or very little. The greatest change was seen in coyotes (Wilson & Shivik, 2011), where a 2.4‐fold higher density was associated with movement rates less than half as fast and home ranges less than a tenth in size. While coyotes exhibited the most dramatic differences in movement rate and home‐range size, it is difficult to generalize the magnitude of these effects across similar taxa. More groundwork needs to be done to assess movement rates of populations at different densities. Because of the variation in effect sizes observed in our meta‐analysis, species or populations that have not been extensively studied should be treated with caution when making inferences about abundance based on CT detection rates.

It is worth reiterating that the nature of telemetry data is to underestimate true movement rates, as longer intervals between relocations will miss fine‐scale movements and hence underestimate speed. In the context of home‐range size, a fast animal that quickly covers its home range is consequently expected to turn frequently (e.g., at boundaries of home range), and may thus appear—when observed at a coarse temporal resolution—to move as slow as, or even slower than, a slower animal traveling along straighter paths within a larger home range. Thus, telemetry studies with longer intervals between relocations are at risk of underestimating the magnitude of density‐dependent changes in movement rates (Street et al., 2018). Most of the telemetry studies included in our meta‐analysis had relatively short fix intervals (≤1 hr; Table S1), and even studies with longer intervals showed the predicted correlation between density and movement (e.g., Panzacchi, Linnell, Odden, Odden, & Andersen, 2009 in Figures 3 and 4, Table S1).

In our simulations, we found that the performance of a detection rate index was influenced by space use. When the assumption of constant space use was upheld and individuals were moving fast over large ranges, the detection rate index performed well. Keeping movement rate and home‐range size constant under such conditions, doubling the abundance approximately doubled the mean detection rate. However, when movement patterns changed with density, the results were less clean. Compared to the fast‐moving, large home‐range scenario, the slower and smaller home‐range population exhibited far greater variation in detection rates (see Neilson et al., 2018 for further discussion of these relationships). While it is encouraging that detection rates did track the general trend in abundance, being significantly different between the low density and high density populations (even under realistic changes in movement), we feel that the large variability introduced by movement behavior undermines the ability of detection rate indices to reliably measure the magnitude of change in relative abundance.

It is also important to note that these simulation results reflect an otherwise ideal set of conditions for camera trapping: Identical individuals moving through a homogeneous landscape with random camera placement and perfect detection. Actual camera trap studies have to contend with additional sources of sampling error. If detection rate indices are nevertheless used to track relative abundance, scientists and managers should keep in mind that the estimated magnitude of the change could be up to 30% smaller than its true value due to density‐dependent movement alone (given a scenario where the population exhibits much slower movement and smaller home‐range sizes at high densities relative to low densities). While this is worrying if the goal is to accurately estimate the magnitude of change in relative abundance, it is nevertheless encouraging that changes in movement are unlikely to totally reverse the trend in detection rate. By encompassing the range in space use found in the meta‐analysis, our simulation likely covers the worst‐case scenario as far as the strength of this particular confounding factor. However, future simulations could expand on this work by exploring the role of additional factors like nonrandom camera placement, density‐dependent functional responses in habitat selection, and more complex movement behaviors (e.g., Van Beest, McLoughlin, Mysterud, & Brook, 2016). Finally, users of these indices should also consider differences in the variance of their estimates, as this may be an earlier indicator of changes in the population, even if mean detection rates are not yet significantly different.

The relationships observed in this analysis could have a significant impact on the outcome of management or conservation decisions. Consider a hypothetical example involving a species of concern which is the target of a novel conservation strategy. Cameras are used to monitor the population response after the implementation of the strategy, with detection rate used as an index of relative abundance. Unfortunately, the conservation strategy fails, and the population declines to an even lower density. At this low density, individuals move faster over larger home ranges—perhaps due to reduced habitat quality and greater difficulty finding patches of good forage. This increases the average encounter rate between individuals and CTs, thus masking the effect of a reduction in the number of individuals on detection rate. An analysis of the camera data might reveal little to no significant change in detection rate after implementation of the strategy. Using detections as a relative abundance index, one might conclude that the novel strategy had successfully slowed or stopped the decline, when in fact it had failed. Therefore, determining the magnitude by which detection rates are affected by movement is a critical step in evaluating the reliability of these indices.

We provided quantitative evidence that across a broad range of taxa home range and movement rate are negatively density‐dependent, violating a core assumption of the use of camera trap detection rates as an index of population abundance. Through simulation analysis, we have shown that the confounding effect of changes in movement and density can reduce the statistical power to detect change in relative abundance from detection data, and that estimates of relative abundance changes may miss up to 30% of the true change. These results support previous calls for caution in the use of relative abundance indices from CT sampling (Burton et al., 2015; Harmsen et al., 2010; Jennelle et al., 2002; Sollmann, Mohamed, et al., 2013). While more statistically sophisticated alternatives are available for estimating density of unmarked populations from camera traps (e.g., Chandler & Royle, 2013; Howe, Buckland, Despres‐Einspenner, & Kuhl, 2017; Moeller, Lukacs, & Horne, 2018; Nakashima, Fukasawa, & Samejima, 2018; Rowcliffe, Field, Turvey, & Carbone, 2008), these require careful planning of study design and entail other assumptions that are largely untested (cf Johnson, 2008). Such methods may require direct measurement, or a priori knowledge, of species movement characteristics, or be similarly susceptible to density‐dependent movement behaviur (e.g., Efford, Dawson, Jhala, & Qureshi, 2016; Rowcliffe, Jansen, Kays, Kranstauber, & Carbone, 2016). These alternatives may thus be currently unattractive to practitioners who chose camera trapping for its simplicity, yet concern has been raised over the continued uncritical application of relative abundance indices to measure population change (Sollmann, Mohamed, et al., 2013). We hope that the evidence presented here will encourage a more cautious consideration of the merits of each analytical method, and more deliberate choice of study design. For example, camera trap detection rates may be suitable for estimating differences in animal activity or use of different habitats, or for general trends in abundance where movement is known to be relatively constant. However, more detailed data on animal movement, and more robust analytical techniques, may be required for stronger inferences on population changes. Given that relative abundance indices are frequently used at the front lines of management and conservation, we urge a more critical look at their use and interpretation, and we call for further research into the reliability of emerging alternative methods to estimate abundance of unmarked populations using camera traps.

## CONFLICT OF INTEREST

None declared.

## AUTHOR CONTRIBUTIONS

All authors conceived the ideas and designed methodology; TA designed the computer simulation; KB analyzed the data and led writing of the initial manuscript; ACB led writing of subsequent drafts. All authors contributed critically to the drafts and gave final approval for publication.

## Data Availability

Data are archived online in the Dryad digital repository (https://datadryad.org).
