# Supplementary material for: Density‐dependent space use affects interpretation of camera trap detection rates
Source: Ecol Evol. 2019 Nov 22;9(24):14031–41. doi: 10.1002/ece3.5840 (PMC6953673; doi:10.1002/ece3.5840)
Supplement: Supplementary file 1 [file ECE3-9-14031-s001.docx]

**Appendix S1:** Supporting information concerning literature review.

**Table S1:** Shortlisted literature from systematic review

| author names | year | order/superorder | common name | species name | density/abundance estimation method | tracking method | home range estimation method | movement fix rate |
| --- | --- | --- | --- | --- | --- | --- | --- | --- |
| **Le Mar and McArthur** | 2005 | australidelphian | brushtail possum | *Trichosurus vulpecula fuliginosus* | secondary | N/A | secondary | N/A |
| **Ashenafi et al.** | 2005 | carnivore | Ethiopian wolf | *Canis simensis* | line transect, secondary | VHF | MCP | N/A |
| **Di Bitetti et al.** | 2006 | carnivore | ocelot | *Leopardus pardalis* | mark-recapture | N/A | secondary | N/A |
| **Stoen et al.** | 2006 | carnivore | brown bear | *Ursus arctos* | survey | VHF | MCP | N/A |
| **St-Pierre et al.** | 2006 | carnivore | ermine | *Mustela erminea* | N/A | VHF | MCP | 12h-24h |
| **St-Pierre et al.** | 2006 | carnivore | long-tailed weasel | *Mustela frenata* | N/A | VHF | MCP | 12h-24h |
| **Astete et al.** | 2008 | carnivore | jaguar | *Panthera onca* | secondary | N/A | secondary | N/A |
| **Jones et al.** | 2008 | carnivore | Western spotted skunk | *Spilogale gracilis amphiala* | MNA | VHF | KDE | N/A |
| **Schmidt** | 2008 | carnivore | Eurasian lynx | *Lynx lynx* | N/A | VHF | MCP | 24h |
| **Wilson and Shivik** | 2011 | carnivore | coyote | *Canis latrans* | secondary | GPS | LoCoH | 15min |
| **Elizalde-Arellano et al.** | 2012 | carnivore | bobcat | *Lynx rufus* | N/A | GPS | MCP, secondary | 1h, secondary |
| **Pereira et al.** | 2012 | carnivore | Geoffroy's cat | *Leopardus geoffroyi* | N/A | VHF | MCP | 30min |
| **Leuchtenberger et al.** | 2013 | carnivore | giant otter | *Pteronura brasiliensis* | secondary | VHF | MCP, KDE, LoCoH | 30min |
| **Allen et al.** | 2014 | carnivore | grey wolf (dingo) | *Canis lupus dingo* | qualitative | GPS | MCP | 30min - 2h |
| **Devillard et al.** | 2008 | lagomorph | European rabbit | *Oryctolagus cuniculus* | unknown | VHF | MCP | N/A |
| **Ganas and Robbins** | 2005 | primate | eastern gorilla | *Gorilla beringei beringei* | N/A | follow | MCP, quadrat | continuous |
| **Merker et al.** | 2005 | primate | Dian's tarsier | *Tarsius dianae* | survey | VHF | MCP | 15min |
| **Stevenson** | 2006 | primate | woolly monkey | *Lagothrix lagothricha* | secondary | follow | MCP | 30min |
| **Cristobal-Azkarate and Arroyo-Rodriguez** | 2007 | primate | mantled howler monkey | *alouatta palliata* | secondary | N/A | secondary | N/A |
| **Irwin** | 2008 | primate | diademed sifaka | *Propithecus diadema* | N/A | follow | MCP, KDE | unknown |
| **Mekonnen et al.** | 2010 | primate | bale monkey | *Chlorocebus djamdjamensis* | N/A | follow | MCP | 15min |
| **Amaral Nascimento et al.** | 2011 | primate | golden lion tamarin | *Leontopithecus rosalia* | secondary | N/A | secondary | secondary |
| **Amaral Nascimento et al.** | 2011 | primate | black-faced lion tamarin | *Leontopithecus caissara* | secondary | follow | MCP, KDE, DMP | 20min |
| **Amaral Nascimento et al.** | 2011 | primate | golden-headed lion tamarin | *Leontopithecus chrysomelas* | secondary | N/A | secondary | secondary |
| **Cristina Palma et al.** | 2011 | primate | red howler monkey | *Alouatta seniculus* | secondary | follow | quadrants, secondary | 15min, secondary |
| **Kim et al.** | 2011 | primate | Javan gibbon | *Hylobates moloch* | N/A | follow | MCP | 30min |
| **Gabriel** | 2013 | primate | ring-tailed lemur | *Lemur catta* | secondary | follow | KDE | continuous |
| **Zhou et al.** | 2014 | primate | Assamese macaque | *Macaca assamensis* | N/A | follow | quadrat | 30min |
| **Schradin and Pillay** | 2005 | rodent | striped mouse | *Rhabdomys pumilio* | mark-recapture | VHF | MCP | N/A |
| **Hoffmann et al.** | 2006 | rodent | African grass rat | *Arvicanthis niloticus* | unknown | VHF | MCP | N/A |
| **Jurczyszyn** | 2006 | rodent | edible dormouse | *Glis glis* | N/A | VHF | MCP | 1h |
| **Arjo et al.** | 2007 | rodent | mountain beaver | *Aplodontia rufa* | MNA | VHF | KDE | N/A |
| **Jurczyszyn and Zgrabczynska** | 2007 | rodent | edible dormouse | *Glis glis* | index | VHF | MCP | 1h |
| **Turrini et al.** | 2008 | rodent | European ground squirrel | *Spermophilus citellus* | MNA | VHF | MCP | N/A |
| **Stradiotto et al.** | 2009 | rodent | yellow-necked mouse | *Apodemus flavicollis* | mark-recapture | VHF | KDE | >=50min |
| **Quirici et al.** | 2010 | rodent | degu | *Octodon degus* | mark-recapture | VHF | MCP, KDE | N/A |
| **Sommaro et al.** | 2010 | rodent | corn mouse | *Calomys musculinus* | known | trapping | MCP | N/A |
| **Lee et al.** | 2012 | rodent | Korean field mouse | *Apodemus peninsulae* | N/A | VHF | MCP | 4.25h |
| **King and Gurnell** | 2005 | ungulate | takhi | *Equus ferus przewalskii* | known | follow | KDE | N/A |
| **McCoy et al.** | 2005 | ungulate | white-tailed deer | *Odocoileus virginianus* | secondary | VHF | KDE | N/A |
| **Brambilla et al.** | 2006 | ungulate | chamois | *Rupicapra rupicapra* | unknown | VHF | MCP | N/A |
| **Webb et al.** | 2007 | ungulate | white-tailed deer | *Odocoileus virginianus* | secondary | VHF | MCP, KDE | N/A |
| **Coulombe et al.** | 2008 | ungulate | white-tailed deer | *Odocoileus virginianus* | known | VHF | N/A | >3h |
| **Neumann et al.** | 2009 | ungulate | moose | *Alces alces* | unknown | GPS | N/A | 30min-1h |
| **Panzacchi et al.** | 2009 | ungulate | roe deer | *Capreolus capreolus* | secondary | VHF | MCP | 24h |

**Appendix S1 References:**

Allen, B.L. & Leung, L.K.-P. (2014) The (Non)Effects of Lethal Population Control on the Diet of Australian Dingoes. *Plos One*, **9**, e108251–e108251.

Amaral Nascimento, A.T., Schmidlin, L.A.J., Valladares-Padua, C.B., Matushima, E.R. & Verdade, L.M. (2011) A Comparison of the Home Range Sizes of Mainland and Island Populations of Black-Faced Lion Tamarins (Leontopithecus caissara) Using Different Spatial Analysis. *American Journal of Primatology*, **73**, 1114–1126.

Arjo, W.M., Huenefeld, R.E. & Nolte, D.L. (2007) Mountain beaver home ranges, habitat use, and population dynamics in Washington. *Canadian Journal of Zoology-Revue Canadienne De Zoologie*, **85**, 328–337.

Ashenafi, Z.T., Coulson, T., Sillero-Zubiri, C. & Leader-Williams, N. (2005) Behaviour and ecology of the Ethiopian wolf (Canis simensis) in a human-dominated landscape outside protected areas. *Animal Conservation*, **8**, 113–121.

Astete, S., Sollmann, R. & Silveira, L. (2008) Comparative ecology of jaguars in Brazil. *CAT News*, 9–14.

Di Bitetti, M.S., Paviolo, A. & De Angelo, C. (2006) Density, habitat use and activity patterns of ocelots (Leopardus pardalis) in the Atlantic Forest of Misiones, Argentina. *Journal of Zoology*, **270**, 153–163.

Brambilla, P., Bocci, A., Ferrari, C. & Lovari, S. (2006) Food patch distribution determines home range size of adult male chamois only in rich habitats. *Ethology Ecology & Evolution*, **18**, 185–193.

Coulombe, M.-L., Cote, S.D. & Huot, J. (2008) Experimental influence of population density and vegetation biomass on the movements and activity budget of a large herbivore. *Behaviour*, **145**, 1167–1194.

Cristina Palma, A., Velez, A., Gomez-Posada, C., Lopez, H., Zarate, D.A. & Stevenson, P.R. (2011) Use of Space, Activity Patterns, and Foraging Behavior of Red Howler Monkeys (Alouatta seniculus) in an Andean Forest Fragment in Colombia. *American Journal of Primatology*, **73**, 1062–1071.

Cristobal-Azkarate, J. & Arroyo-Rodriguez, V. (2007) Diet and activity pattern of howler monkeys (Alouatta palliata) in Los Tuxtlas, Mexico: Effects of habitat fragmentation and implications for conservation. *American Journal of Primatology*, **69**, 1013–1029.

Devillard, S., Aubineau, J., Berger, F., Leonard, Y., Roobrouck, A. & Marchandeau, S. (2008) Home range of the European rabbit (Oryctolagus cunicalus) in three contrasting French populations. *Mammalian Biology*, **73**, 128–137.

Elizalde-Arellano, C., Carlos Lopez-Vidal, J., Hernandez, L., Laundre, J.W., Cervantes, F.A. & Alonso-Spilsbury, M. (2012) Home Range Size and Activity Patterns of Bobcats (Lynx rufus) in the Southern Part of their Range in the Chihuahuan Desert, Mexico. *American Midland Naturalist*, **168**, 247–264.

Gabriel, D.N. (2013) Habitat Use and Activity Patterns as an Indication of Fragment Quality in a Strepsirrhine Primate. *International Journal of Primatology*, **34**, 388–406.

Ganas, J. & Robbins, M.M. (2005) Ranging behavior of the mountain gorillas (Gorilla beringei beringei) in Bwindi Impenetrable National Park, Uganda: a test of the ecological constraints model. *Behavioral Ecology and Sociobiology*, **58**, 277–288.

Hoffmann, A., Eckhoff, K. & Klingel, H. (2006) Spatial and temporal patterns in Arvicanthis niloticus (Desmarest, 1822) as revealed by radio-tracking. *African Journal of Ecology*, **44**, 72–76.

Irwin, M.T. (2008) Diademed sifaka (Propithecus diadema) ranging and habitat use in continuous and fragmented forest: Higher density but lower viability in fragments? *Biotropica*, **40**, 231–240.

Jones, K.L., Van Vuren, D.H. & Crooks, K.R. (2008) Sudden increase in a rare endemic carnivore: Ecology of the island spotted skunk. *Journal of Mammalogy*, **89**, 75–86.

Jurczyszyn, M. (2006) The use of space by translocated edible dormice, Glis glis (L.), at the site of their original capture and the site of their release: Radio-tracking method applied in a reintroduction experiment. *Polish Journal of Ecology*, **54**, 345–350.

Jurczyszyn, M. & Zgrabczynska, E. (2007) Influence of population density and reproduction on space use and spatial relations in the edible dormouse. *Acta Theriologica*, **52**, 181–188.

Kim, S., Lappan, S. & Choe, J.C. (2011) Diet and Ranging Behavior of the Endangered Javan Gibbon (Hylobates moloch) in a Submontane Tropical Rainforest. *American Journal of Primatology*, **73**, 270–280.

King, S.R.B. & Gurnell, J. (2005) Habitat use and spatial dynamics of takhi introduced to Hustai National Park, Mongolia. *Biological Conservation*, **124**, 277–290.

Lee, E.J., Rhim, S.-J. & Lee, W.-S. (2012) Seasonal Movements and Home Range Sizes of Korean Field Mouse Apodemus peninsulae in Unburned and Post-Fire Pine Planted Stands Within a Pine Forest. *Journal of Animal and Veterinary Advances*, **11**, 3834–3839.

Leuchtenberger, C., Rodrigues Oliveira-Santos, L.G., Magnusson, W. & Mourao, G. (2013) Space use by giant otter groups in the Brazilian Pantanal. *Journal of Mammalogy*, **94**, 320–330.

Le Mar, K. & McArthur, C. (2005) Habitat selection by common brushtail possums in a patchy eucalypt-forestry environment. *Australian Mammalogy*, **27**, 119–127.

McCoy, J.E., Hewitt, D.G. & Bryant, F.C. (2005) Dispersal by yearling male white-tailed deer and implications for management. *Journal of Wildlife Management*, **69**, 366–376.

Mekonnen, A., Bekele, A., Fashing, P.J., Hemson, G. & Atickem, A. (2010) Diet, Activity Patterns, and Ranging Ecology of the Bale Monkey (Chlorocebus djamdjamensis) in Odobullu Forest, Ethiopia. *International Journal of Primatology*, **31**, 339–362.

Merker, S., Yustian, I. & Muhlenberg, M. (2005) Responding to forest degradation: altered habitat use by Dian’s tarsier Tarsius dianae in Sulawesi, Indonesia. *Oryx*, **39**, 189–195.

Neumann, W., Ericsson, G. & Dettki, H. (2009) The non-impact of hunting on moose Alces alces movement, diurnal activity, and activity range. *European Journal of Wildlife Research*, **55**, 255–265.

Panzacchi, M., Linnell, J.D.C., Odden, M., Odden, J. & Andersen, R. (2009) Habitat and roe deer fawn vulnerability to red fox predation. *Journal of Animal Ecology*, **78**, 1124–1133.

Pereira, J.A., Walker, R.S. & Novaro, A.J. (2012) Effects of livestock on the feeding and spatial ecology of Geoffroy’s cat. *Journal of Arid Environments*, **76**, 36–42.

Quirici, V., Castro, R.A., Ortiz-Tolhuysen, L., Chesh, A.S., Burger, J.R., Miranda, E., Cortes, A., Hayes, L.D. & Ebensperger, L.A. (2010) Seasonal variation in the range areas of the diurnal rodent Octodon degus. *Journal of Mammalogy*, **91**, 458–466.

Schmidt, K. (2008) Behavioural and spatial adaptation of the Eurasian lynx to a decline in prey availability. *Acta Theriologica*, **53**, 1–16.

Schradin, C. & Pillay, N. (2005) Intraspecific variation in the spatial and social organization of the African striped mouse. *Journal of Mammalogy*, **86**, 99–107.

Sommaro, L. V, Steinmann, A.R., Chiappero, M.B. & Priotto, J.W. (2010) Effect of high density on the short term Calomys musculinus spacing behaviour: A fencing experiment. *Acta Oecologica-International Journal of Ecology*, 36, 343–348.

Stevenson, P.R. (2006) Activity and ranging patterns of Colombian woolly monkeys in north-western Amazonia. *Primates*, **47**, 239–247.

Stoen, O.G., Zedrosser, A., Saebo, S. & Swenson, J.E. (2006) Inversely density-dependent natal dispersal in brown bears Ursus arctos. *Oecologia*, **148**, 356–364.

St-Pierre, C., Ouellet, J.-P. & Crete, M. (2006) Do competitive intraguild interactions affect space and habitat use by small carnivores in a forested landscape? *Ecography*, **29**, 487–496.

Stradiotto, A., Cagnacci, F., Delahay, R., Tioli, S., Nieder, L. & Rizzoli, A. (2009) Spatial Organization of the Yellow-Necked Mouse: Effects of Density and Resource Availability. *Journal of Mammalogy*, **90**, 704–714.

Turrini, T.A., Brenner, M., Millesi, E. & Hoffmann, I.E. (2008) Home ranges of European Ground Squirrels (Spermophilus citellus) in two habitats exposed to different degrees of human impact. *Lynx (Prague)*, **39**, 323–332.

Webb, S.L., Hewitt, D.G. & Hellickson, M.W. (2007) Scale of management for mature male white-tailed deer as influenced by home range and movements. *Journal of Wildlife Management*, **71**, 1507–1512.

Wilson, R.R. & Shivik, J.A. (2011) Contender pressure versus resource dispersion as predictors of territory size of coyotes (Canis latrans). *Canadian Journal of Zoology-Revue Canadienne De Zoologie*, **89**, 960–967.

Zhou, Q., Wei, H., Huang, Z., Krzton, A. & Huang, C. (2014) Ranging behavior and habitat use of the Assamese macaque (Macaca assamensis) in limestone habitats of Nonggang, China. *Mammalia*, **78**, 171–176.
